# Evaluation of the Sidekick point-of-care progesterone lateral-flow assay for use in equine reproductive management

**DOI:** 10.1177/10406387261460834

**Published:** 2026-07-08

**Authors:** Sara H. McDowell, Andrew McLain, Luca Moore, Craig J. Ledgerwood, Elizabeth Bailie, Tara Moore

**Affiliations:** Future Medicines Institute, Ulster University, Belfast, Northern Ireland, United Kingdom; Future Medicines Institute, Ulster University, Belfast, Northern Ireland, United Kingdom; University of York, Heslington, York, United Kingdom; Future Medicines Institute, Ulster University, Belfast, Northern Ireland, United Kingdom; Future Medicines Institute, Ulster University, Belfast, Northern Ireland, United Kingdom; Future Medicines Institute, Ulster University, Belfast, Northern Ireland, United Kingdom

**Keywords:** equine reproduction, lateral-flow immunoassay, progesterone, point-of-care testing

## Abstract

A point-of-care (POC; stall-side) test for progesterone that delivers rapid, accurate results has the potential to transform equine reproductive care. We evaluated the analytical and clinical performance of a POC device (Sidekick) that incorporates a progesterone lateral-flow assay (LFA) with a reader, and compared it with established methods (radioimmunoassay [RIA] and chemiluminescence immunoassay [CLIA]). Analytical performance was assessed using serially diluted mare plasma (0–8 ng/mL progesterone) to estimate precision and percent recovery. Clinical accuracy was assessed by comparison with RIA and CLIA on 19 paired serum samples. Agreement was evaluated by regression analyses and ordinal concordance across clinically relevant progesterone bands. The LFA had excellent recovery (90–102%) across 4–8 ng/mL, strong repeatability (CV 4–15%), and high reproducibility between 2 laboratories (CV 9.9%). Ordinal agreement with reference standards was near-perfect in spiked samples (κ = 0.94) and substantial in clinical samples versus both RIA (κ = 0.77) and CLIA (κ = 0.79). Cross-reactivity with the synthetic progestin, altrenogest, was tested at pharmacologic concentrations; no cross-reactivity was detected. Limitations of our study include an observed bias, potentially caused by the matrix differing between methods (serum vs. plasma). Additionally, a narrow validated range could cause underestimation of high concentrations. The LFA in the Sidekick device provided rapid, accurate, and clinically meaningful progesterone results in mares across 4–8 ng/mL, which would enable on-the-spot decisions and avoid delays from sample processing, transport, and result reporting.

Accurate monitoring of serum progesterone concentrations is beneficial for reproductive management in mares because it can confirm luteal activity, identify luteal insufficiency, and monitor high-risk pregnancies.^12,20^ Most embryonic losses occur in early pregnancy when the corpus luteum is the main source of progesterone^12^; reduced concentrations of progesterone in early pregnancy are associated with increased early embryonic losses.^18^ If progesterone is low at the initial pregnancy diagnosis, then a progestogen can be administered to prevent loss of pregnancy,^10,15^ which is of particular importance in mares with a history of pregnancy loss, urinary tract infections, and luteal insufficiency.^13–15^ Timely and precise assessment of the progesterone concentration is often essential for making informed decisions on whether to initiate or continue progestin treatment. An equine progesterone test should specifically detect the native progesterone produced by the corpus luteum and not cross-react with progestin treatment.^20^

The current gold standard for progesterone measurement is laboratory-based immunoassay, including radioimmunoassay (**RIA**) and chemiluminescence immunoassay (**CLIA**), which offer high sensitivity and specificity^5,7^ and do not cross-react with altrenogest (progestin).^20^ Limitations of RIA and CLIA are that they require specialized instrumentation, laboratory infrastructure, skilled technical personnel, and sample processing; transport can introduce delays in test turnaround time.^6^ These limitations are particularly relevant in ambulatory or field-based equine practice, where access to laboratory services may be restricted.

Point-of-care (**POC**) testing technologies have been developed to enable rapid, POC (stall-side) hormone analysis, and can offer clinicians actionable results within minutes.^3,4,9^ POC testing is a proven tool for veterinarians in time-sensitive applications, such as the detection of serum amyloid A (a marker of inflammation)^11,17^ and equine influenza virus.^2^ Before a POC progesterone assay can be recommended for routine clinical use on mare samples, it must be evaluated for analytical performance, including linearity, precision, and accuracy in comparison with the validated reference methods (i.e., RIA and CLIA), to establish diagnostic reliability.^16,19^

Here, we report our assessment of the analytical performance of the progesterone lateral-flow assay (**LFA**) in the POC lateral-flow device (**LFD**; Sidekick Animal Health), a hand-held device that incorporates a LFA with an integral reader. Each cartridge contains the LFA and is used for one test per sample. The device is calibrated automatically (not by the user). An individual test is read against a standard curve that is downloaded and updated automatically upon opening the associated smartphone application and pairing the reader. We used a single device for the testing in our study.

## Materials and methods

Blood sample collection was carried out by a registered veterinarian during the first veterinary consultation to confirm pregnancy. All samples used in our study were frozen remnant serum or plasma samples collected at the same time from each mare. Our study was retrospective; therefore, it was not necessary to seek ethics board approval. Owner consent had been obtained for the animals used in our study. Sample collection followed the “Diagnostic Sample Collection, Handling, and Transport Guide” set by the American Association of Equine Practitioners, 2024.^1^

The 19 mares used in our study were >3-y-old Thoroughbreds from Ireland (counties Wicklow and Kildare), pregnant in the embryonic period (“first trimester”; confirmed by ultrasound) and ≥14 d since artificial insemination or mating with a stallion. No >3 mares were from the same stable. We tested the progesterone LFA kit with samples for embryonic period mares only.

A plasma sample donated from a pregnant mare (40 d, 17 ng/mL) was serially diluted in progesterone-free plasma using commercial gelding donor plasma (Lampire Biological) to create reference standards that spanned the clinically relevant range in breeding mares (0, 2, 4, 6, 8 ng/mL progesterone), for use in linearity and precision measurements of the LFA. Using the LFD, 5 replicates were assessed for each progesterone plasma standard (0, 2, 4, 6, 8 ng/mL) by a separate individual at 2 academic laboratories (referred to as Labs 1 and 2) in Ulster University campus locations (Belfast, Northern Ireland, UK).

The LFA was performed for the 19 equine plasma samples, in duplicate, at Labs 1 and 2. RIA (single measurement) and CLIA (duplicate) were performed on 19 paired serum samples, with all analyses carried out under standard laboratory conditions.

### Progesterone LFA

The progesterone LFA kit was used according to the manufacturer’s instructions. In brief, each sample was mixed in a 1:1 ratio with dilution buffer. After mixing, 130 µL were added directly to the LFD, which was left at room temperature and allowed to react for 5.0 min. After 5.0 min, the signal intensity of the LFA was read automatically on the integral reader, which outputs the corresponding progesterone concentration from a preprogrammed calibration curve. Raw data from the LFD reader was captured as evidence of completion by photographing the result displayed on the reader. Additionally, raw data were stored on the connected LFDs.

### Progesterone RIA

Sera were tested by RIA (0717010-CF; MP Biomedicals) at the Animal Health Diagnostic Centre, College of Veterinary Medicine, Cornell University (Ithaca, NY, USA), according to the manufacturer’s instructions. The progesterone RIA had been validated internally for use on equine serum samples. Within each run, a standard curve is generated using standards included in the RIA kit. The calculated sensitivity of the assay is 0.06 ng/mL. The mean intra- and inter-assay CVs were 6.3 and 8.7%, respectively.

### Progesterone CLIA

Sera were tested by CLIA (Immulite progesterone assay; Siemens) in Ulster University campus locations (Belfast, Northern Ireland, UK), according to the manufacturer’s instructions. This CLIA has been used with equine serum samples and in commercial laboratories.^20^ The instrument print-out was kept as evidence of results. The analyzer was calibrated for each test kit using the supplied adjustor samples.

### Progestin cross-reactivity assessment

To determine if the LFA detects synthetic progestin, altrenogest (SKV222; Merck) was first dissolved to a concentration of 1 mg/mL in pure ethyl alcohol (459836; Merck) and then diluted to 1 µg/mL in progesterone-free plasma (gelding donor). Three samples were tested in triplicate with the LFA to assess cross-reactivity.

### Data analysis

Linearity was evaluated by percent-recovery per level and ANOVA lack-of-fit. Precision was reported as repeatability (within-site CV%) and reproducibility (between-site CV%) with 95% CIs. The CIs were obtained by nonparametric bootstrap testing (5,000 resamples). Values below the level of quantification (**LOQ**) were excluded from the analysis. When <80% of replicates for the reference group were above the LOQ, the entire group was excluded. For agreement analysis only, values <LOQ were inputted as “√*LOQ” to maximize retention of samples across the measurement range*.

Agreement between the LFA and the RIA and CLIA reference methods was assessed with Deming regression (variance ratio λ estimated from replicate SDs; bootstrap 95% CIs) and Bland–Altman (bias, 95% level of agreement [**LOA**]; proportional-bias check). Pearson *r* is descriptive only. Deming regression was used to compare the 2 methods and to account for errors in both methods; the bootstrap method was used to determine robust CIs for slope and intercept. Bland–Altman analysis was used to illustrate the difference between paired readings against their means. Ordinal agreement between the LFA and the RIA and CLIA reference methods was assessed across 5 clinically defined progesterone bands (<3, 3 to <4, 4 to <6, 6 to <8, ≥8 ng/mL), which reflect decision thresholds for luteal sufficiency. Values <3 ng/mL were retained within the <3 ng/mL band to avoid censoring near the lower end of the range. Agreement was summarized using quadratic-weighted Cohen κ, which awards partial credit for adjacent-band disagreements (i.e., misclassifying 3.9 as 4.1 is penalized less than 3.9 as ≥8). Statistical analysis was completed using R (v.4.4.2) statistical programming language.

## Results

### Linearity and precision assessment

Linearity was assessed to ensure that the LFA response was proportional to hormone concentration across a clinically relevant range. The linearity of the LFA was first evaluated by testing known progesterone-positive mare plasma samples (determined by CLIA and RIA) at known concentrations of progesterone (4, 6, 8 ng/mL) with 5 runs per concentration at Labs 1 and 2 (*n* = 30 combined). Across both laboratories, the mean recoveries were 92.5% at 4 ng/mL (mean 3.7 ng/mL; 95% CI [3.4, 4.0]), 102% at 6 ng/mL (6.1 ng/mL; 95% CI [5.7, 6.5]), and 90% at 8 ng/mL (7.2 ng/mL; 95% CI [6.8, 7.7]). The mean recoveries in the LFA were compared with the known concentration of the reference samples (**Table 1**).

**Table 1.** Linearity and precision of the progesterone lateral-flow assay for progesterone plasma reference samples across 2 academic laboratories.

Progesterone reference concentration, ng/mL	Average reported concentration, ng/mL [95% CI]	Lab 1 intra-assay variation, CV%	Lab 2 intra-assay variation, CV%	Inter-assay variation, CV%	*p*-value
4	3.7 [3.35, 4.05]	11.8	15.2	10.7	0.62
6	6.1 [5.69, 6.51]	3.7	14.4	13.0	0.4
8	7.2 [6.75, 7.65]	4.1	11.6	5.92	0.61
Mean %CV		6.5	13.7	9.86	

For the precision assessment, we examined the reproducibility of the test under consistent conditions. The intra-assay precision was determined by comparing the reported mean recovery to the known progesterone concentration at both test laboratories (Table 1). Intra-assay variation was measured using CV% of each of the 4, 6, and 8 ng/mL standards at each site, but it was not possible to measure the CV for the 0 and 2 ng/mL standards because each gave ≥1 reading below the LOQ. The mean CV of intra-assay variation at both laboratories was 6.5 and 13.7% (Table 1).

Inter-assay variation was used to measure variation in results between the 2 laboratories (Table 1). The overall mean inter-assay CV was 9.86%. No statistically significant difference was observed between the progesterone concentration measured using a Sidekick LFD (same device in each lab) for each standard at either Lab 1 or 2, suggesting that this assay was robust at both test laboratories.

**Figure 1.**
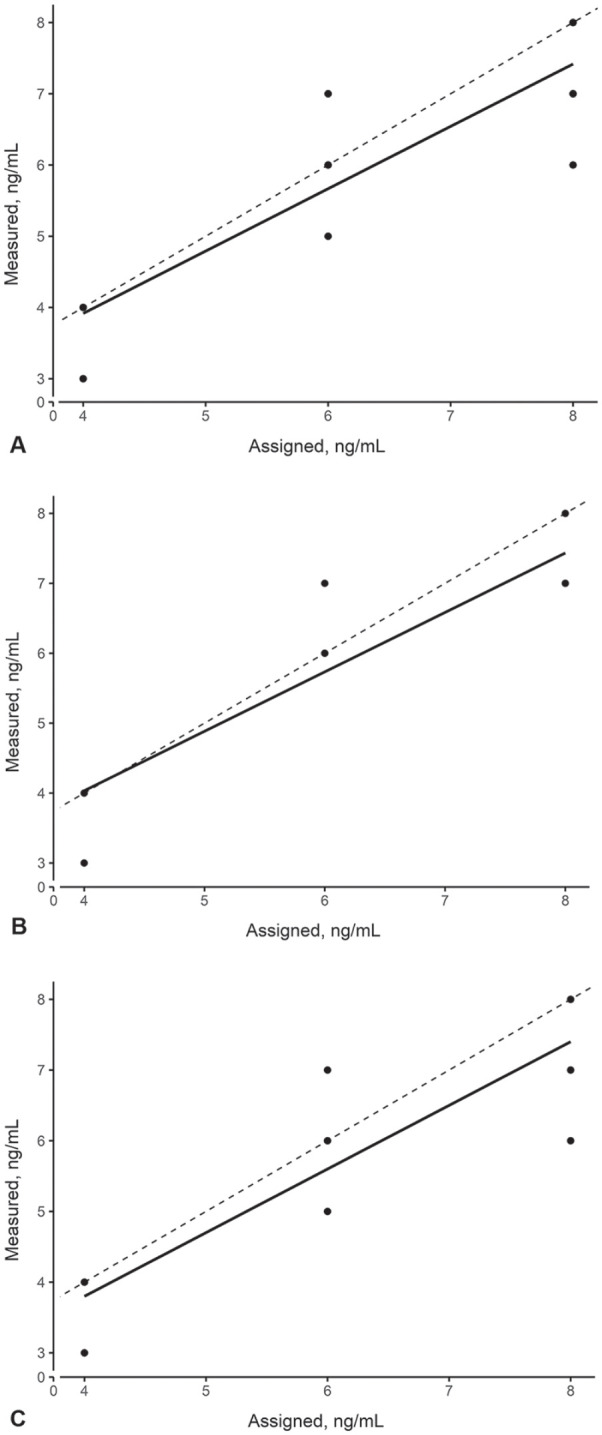
Evaluation of precision and model fit using an ANOVA lack-of-fit test for measured versus assigned progesterone concentrations across 2 laboratories. **A.** Combined plot by laboratory of measured concentration (LFA, plasma) versus assigned concentrations (4, 6, 8 ng/mL). Points are individual runs (5 per concentration per lab); the dashed line is the line of identity (*y* = *x*). Solid lines are ordinary least-squares fits for each laboratory, illustrating good overall linearity with visible under-recovery at 8 ng/mL. **B.** Lab 1 only: linear fit relative to identity; ANOVA lack-of-fit was significant (F(1,12) = 8.17; *p* = 0.014), driven by under-recovery at 8 ng/mL. **C.** Lab 2 only: linear fit relative to identity; lack-of-fit was not significant (F(1,12) = 2.40; *p* = 0.147). Together, these data support a validated semi-quantitative reportable range of 4–8 ng/mL.

An ANOVA lack-of-fit test on the combined dataset from both test laboratories indicated deviation from strict linearity (F(1,27) = 8.84; *p* = 0.006), driven by under-recovery at 8 ng/mL and slight over-recovery near 6 ng/mL. By site, lack-of-fit was present in Lab 1 (F(1,12) = 8.17; *p* = 0.014) but not in Lab 2 (F(1,12) = 2.40, *p* = 0.147; **Fig. 1**). These results support a validated reportable range of 4–8 ng/mL for semi-quantitative use. Across spiked standards (0, 2, 4, 6, 8 ng/mL; 5 replicates per level in 2 laboratories), the LFA had near-perfect ordinal agreement with the reference: κ = 0.94 (95% CI [0.90, 0.96]). Disagreements were rare and, when present, occurred almost exclusively between adjacent bands surrounding the 3–4 ng/mL decision region (**Table 2**).

**Table 2.** Ordinal agreement between a point-of-care lateral-flow assay (LFA) and progesterone reference standards.

LFA, ng/mL	Progesterone reference concentration, ng/mL				
	<3	3 to <4	4 to <6	6 to <8	≥8
<3	17	0	0	0	0
3 to <4	3	0	3	0	0
4 to <6	0	0	7	1	0
6 to <8	0	0	0	9	7
≥8	0	0	0	0	3

### Comparison with RIA and CLIA

**Figure 2.**
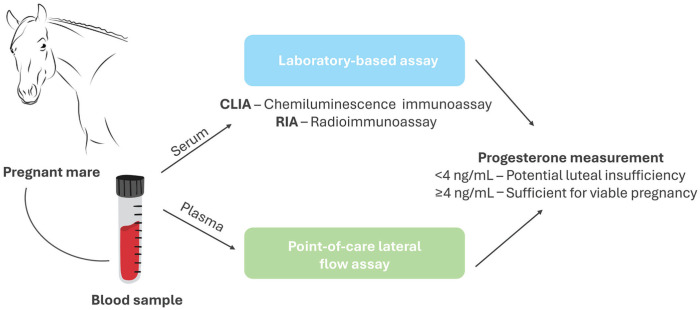
Comparison of progesterone measurement between gold-standard laboratory-based methods (radioimmunoassay and chemiluminescence immunoassay) and a point-of-care lateral-flow assay. Progesterone measurement of <4 ng/mL could indicate potential luteal insufficiency with intervention required, whereas ≥4 ng/mL is an indicator of sufficiency for a viable pregnancy. Figure created with free-to-use illustrations from pixabay and sketchify at Canva.com.

Nineteen paired clinical specimens were analyzed with 3 assays: LFA, CLIA, and RIA (**Suppl. Table 1**). LFA testing was performed in duplicate on plasma. CLIA and RIA were performed on serum from the 19 paired clinical sera for comparison (**Fig. 2**).

For the method comparison, per-sample means were used. Given that 2 matrices (plasma vs. serum) were used across the 3 assays, agreement estimates were interpreted with this caveat in mind. To stringently compare the methods for progesterone detection, the agreement between the 2 methods was quantified using Deming regression with bootstrap CIs and Bland–Altman analysis, first comparing LFA to RIA and then LFA to CLIA.

**Figure 3.**
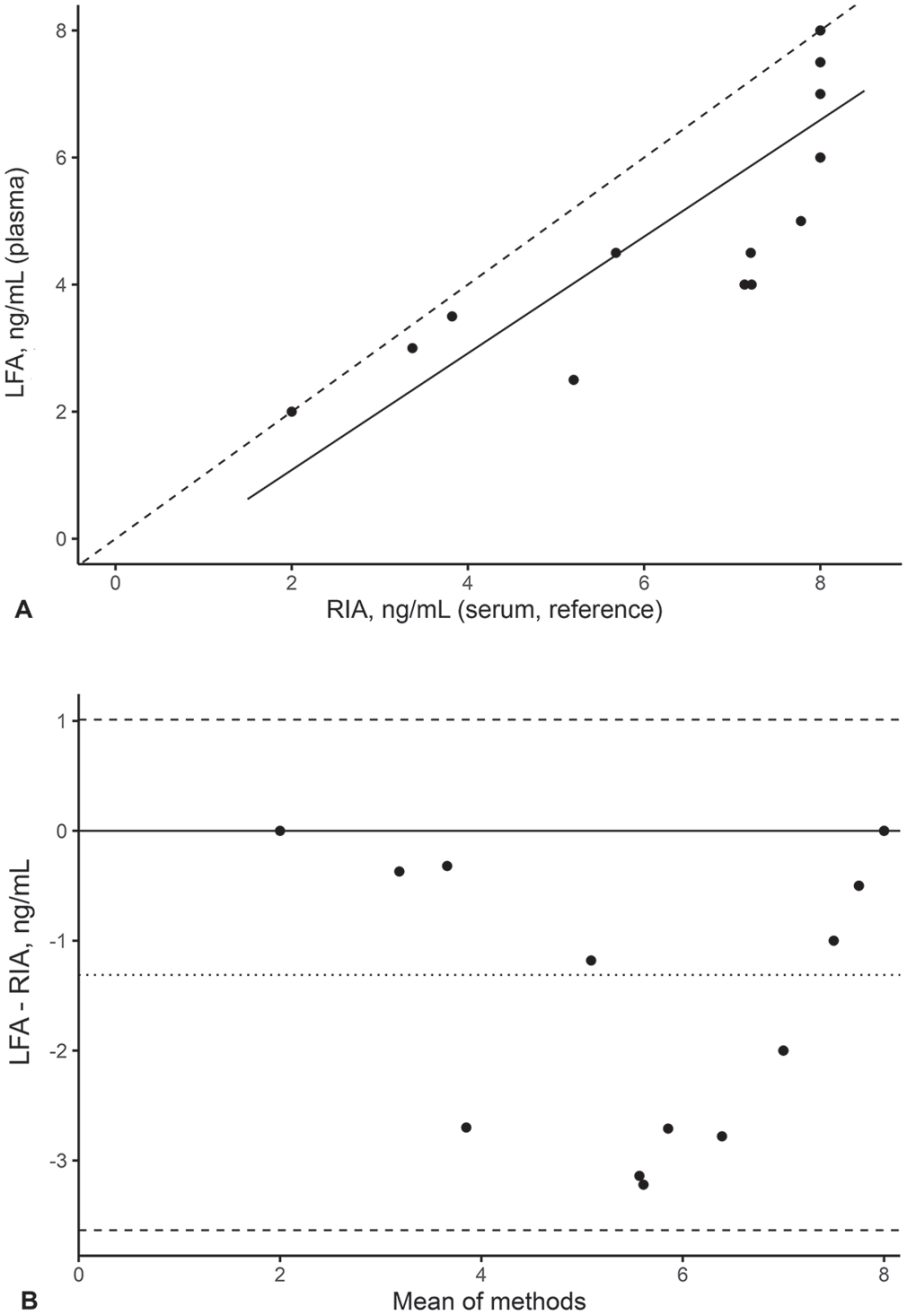
Method comparison of progesterone concentrations using Deming regression and Bland–Altman analysis between a lateral-flow assay (LFA) and radioimmunoassay (RIA). **A.** Deming regression of LFA (plasma) against RIA (serum; *n* = 19 paired samples). Solid line is the Deming fit (slope 0.92, 95% CI [0.72, 1.81]; intercept −0.75 ng/mL, 95% CI [−7.43, 0.30]); dashed line is identity (y = x). λ (error-variance ratio, σ²y/σ²x*) estimated from replicates was 6.32 (RIA single replicate; RIA SD assumed small). **B.** Bland–Altman plot of the difference (LFA−RIA) versus the mean of methods. Dotted line indicates mean bias (−1.31 ng/mL); dashed lines are 95% limits of agreement [−3.64, 1.01 ng/mL]. Matrix difference (plasma vs. serum) may contribute to bias.

RIA provides a single measurement per sample; therefore, the measurement-error ratio (λ) for LFA vs. RIA was estimated from LFA replicate variability together with a conservative assumed RIA SD, yielding λ = 6.32. Against RIA, LFA results had a Deming slope of 0.92 (95% CI [0.72, 1.81]) and an intercept of −0.75 ng/mL (95% CI [−7.43, 0.30]), meaning that as the concentration of progesterone increased, LFA results tended to be slightly lower than RIA results. However, this bias was not statistically significant, and a strong correlation between the 2 methods was observed (**Fig. 3A**). Bland–Altman analysis indicated a mean bias (LFA−RIA) of −1.31 ng/mL with 95% LOAs [−3.64, 1.01 ng/mL]. This was consistent with a small negative average bias and a slope near unity over the observed range, as seen in the Deming regression analysis (**Fig. 3B**).

**Figure 4.**
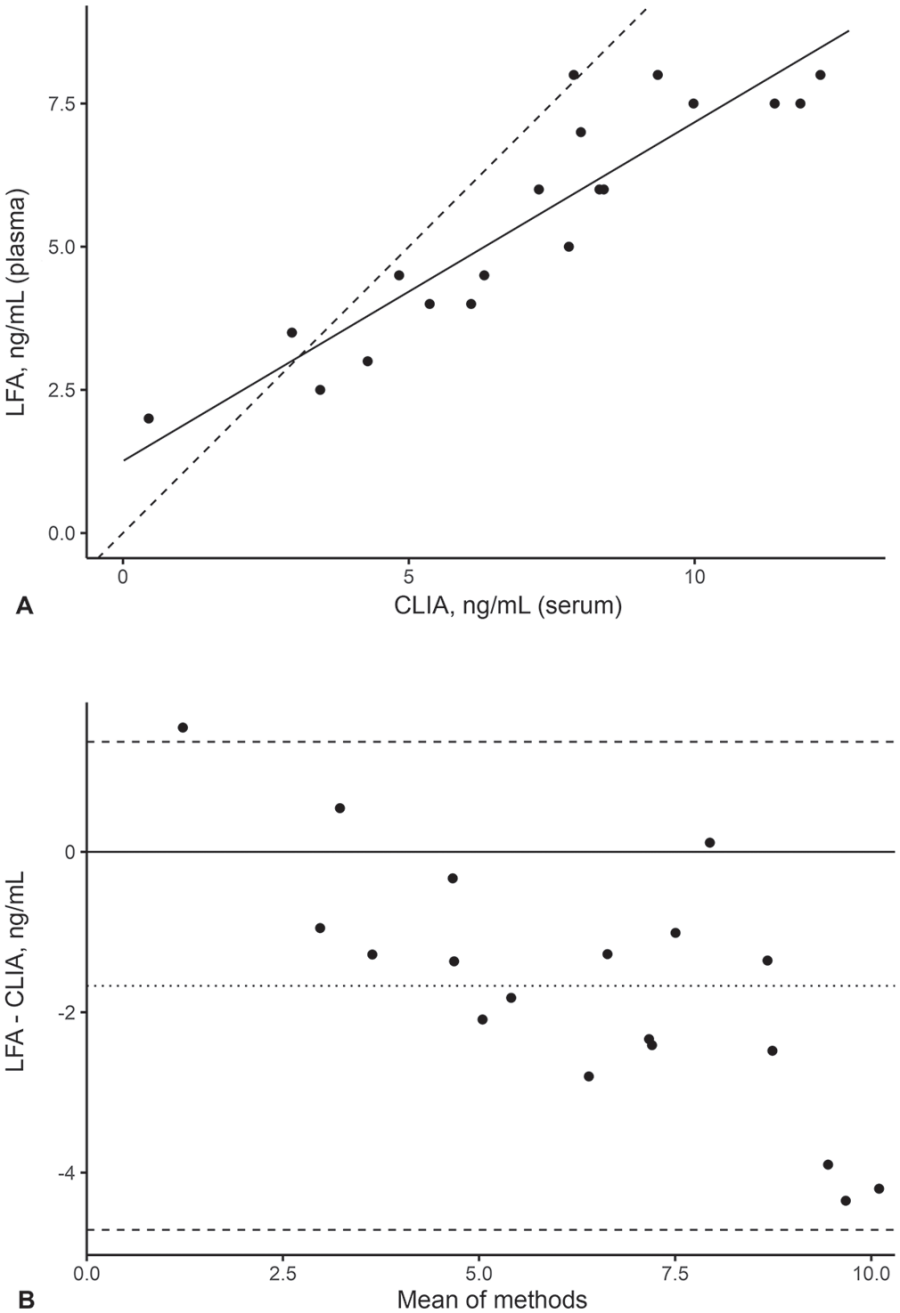
Method comparison of progesterone concentrations using Deming regression and Bland–Altman analysis between lateral-flow assay (LFA) and chemiluminescence immunoassay (CLIA). **A.** Deming regression of LFA (plasma) against CLIA (serum; *n* = 19 paired samples). Solid line is the Deming fit (slope 0.592, 95% CI 0.514–0.746; intercept 1.26 ng/mL, 95% CI 0.22–1.92); dashed line is identity (y = x). λ estimated from replicate SDs was 3.19. **B.** Bland–Altman plot of the difference (LFA − CLIA) versus the mean of methods. Dotted line indicates mean bias (−1.67 ng/mL); dashed lines show 95% limits of agreement (−4.71, 1.37 ng/mL). Values indicate a proportional difference (slope <1), with the LFA lower at higher concentrations.

Compared with the CLIA, LFA results had a proportional difference. Using λ estimated from replicate variability of LFA and CLIA (λ = 3.19), the Deming slope was 0.592 (95% CI [0.514, 0.746]) with an intercept of 1.26 ng/mL (95% CI [0.22, 1.92]), meaning that despite a strong linear relationship between the 2 methods, LFA results were significantly lower than the CLIA results at higher progesterone concentrations (**Fig. 4A**). Bland–Altman analysis gave a mean bias (LFA − CLIA) of −1.67 ng/mL with 95% LOA [−4.71, 1.37 ng/mL], indicating that LFA results were slightly lower than CLIA results at higher concentrations, which was consistent with the Deming regression analysis (**Fig. 4B**).

A λ-sensitivity analysis was conducted to assess the robustness of the Deming regression and to provide confidence in the conclusions. The data-driven λ used in the primary analysis was 6.32 for LFA vs. RIA (RIA single replicate; RIA SD assumed small) and 3.19 for LFA vs. CLIA (from replicate SDs). The λ-sensitivity analysis confirmed that, although the LFA-vs.-RIA slope varied modestly with the assumed-error ratio, the qualitative conclusions were unchanged. The LFA had a small negative average bias compared with RIA, with proportional agreement close to unity. The LFA had a clear proportional difference compared with CLIA, irrespective of λ (**Suppl. Table 2**).

To further investigate the agreement between the LFA and RIA, as well as the LFA and CLIA, we carried out an ordinal agreement analysis between the methods across 5 clinically relevant progesterone bands (Table 2). The ordinal agreement was substantial for both comparisons: κ = 0.77 (95% CI [0.56, 0.89]) for LFA vs. RIA and κ = 0.79 (95% CI [0.55, 0.88]) for LFA vs. CLIA (*n* = 19 for each). Misclassifications were concentrated at band boundaries, consistent with expected variability near decision thresholds. In both comparisons, LFA tended to classify a 6 to <8 ng/mL progesterone band, whereas RIA and CLIA classified this as >8 ng/mL progesterone (**Table 3**).

**Table 3.** Ordinal agreement between point-of-care lateral flow assay (LFA) and RIA and CLIA laboratory comparators.

LFA, ng/mL	RIA, ng/mL					CLIA, ng/mL				
	<3	3 to <4	4 to <6	6 to <8	≥8	<3	3 to <4	4 to <6	6 to <8	≥8
<3	1	0	1	0	0	1	1	0	0	0
3 to <4	0	2	0	0	0	1	0	1	0	0
4 to <6	0	0	1	4	0	0	0	2	3	0
6 to <8	0	0	0	0	7	0	0	0	1	6
≥8	0	0	0	0	3	0	0	0	1	2

### Cross-reactivity with synthetic progestin

To assess the cross-reactivity of the LFA with a synthetic progestin, altrenogest was spiked into equine plasma to a concentration of 1 μg/mL. No cross-reactivity was observed, given that, in all cases, the LFA was <2 ng/mL (**Suppl. Table 3**).

## Discussion

We found that the LFA results were repeatable and decision-useful across 4–8 ng/mL, with recoveries of 92%, 102%, and 90% at 4, 6, and 8 ng/mL, respectively, and within-site CVs of 4–15%. A modest but statistically significant lack-of-fit was observed overall, driven by under-recovery at 8 ng/mL, which can be a limitation of capillary-flow immunoassays at higher analyte concentrations.^8^

When benchmarked against laboratory methods, the POC LFA behaved as expected for a first-line screening test. Relative to RIA, Deming regression and Bland–Altman analysis indicated that the LFA readings tended to be lower than RIA by ~1 ng/mL, with proportional agreement close to unity across the observed range. Compared with the CLIA, the LFA result was progressively lower at higher concentrations. Importantly, RIA had one replicate per sample; therefore, the measurement-error ratio was estimated using LFA replicates, assuming a small RIA SD, and profiled using a wide range of λ values. The λ-sensitivity analysis was stable across plausible error ratios; comparisons of LFA to RIA and to CLIA were robust and did not change as a result of error ratio assumptions.

Given that LFA is semi-quantitative, it was of clinical importance to determine whether progesterone concentrations measured were assigned to the correct concentration band. This is especially important for 3 to <4 ng/mL, because progesterone concentrations <4 ng/mL could be considered as luteal insufficient and would guide progestin supplementation decisions.^12,15,18^ In ordinal agreement analysis, the LFA result almost always agreed with the progesterone reference standard at the band level; when it did not, the reading typically was off by just one neighboring band. This was further corroborated by the comparative study of the LFA to RIA and CLIA, in which the ordinal agreement supported the intended semi-quantitative use of the LFA. For the 19 clinical samples tested, 3 samples had concentrations of <4 ng/mL, as measured by RIA and CLIA, and the LFA also classified these as <4 ng/mL.

Extending our study to increase sample size and focusing on a clinically determined threshold may allow for a more thorough evaluation of clinical performance. However, the LFA appears to be well-suited to triage and can measure progesterone states reliably at 4–8 ng/mL. In settings in which a single value near a cutoff would alter management, a pragmatic approach would be to pair the POC result with either 1) method-specific action thresholds that account for the observed bias, or 2) reflex laboratory confirmation when results fall in a “borderline” zone.

Several analytical details temper interpretation. First, the matrix differed between methods (LFA on plasma; RIA and CLIA on serum), which may contribute to part of the observed bias. Second, the validated range was narrow; under-recovery at 8 ng/mL indicates that very high concentrations may be underestimated; however, this was not of clinical significance. Values <4 ng/mL should be treated as detected or not-detected rather than as precise quantities. Third, the single-replicate design for RIA required an assumption about its short-term variability. Although the sensitivity analysis was reassuring, matched-replicate reference testing would further tighten estimates.

Operationally, a POC device offers advantages that extend beyond analytics: minimal training, no cold-chain transport, immediate communication with owners, and enabling real-time decision-making. Those benefits may justify the loss of precision relative to laboratory immunoassays, provided reporting reflects the semi-quantitative nature of the LFA and users are guided on when to confirm results.

## Supplemental Material

sj-pdf-1-vdi-10.1177_10406387261460834 – Supplemental material for Evaluation of the Sidekick point-of-care progesterone lateral-flow assay for use in equine reproductive management Supplemental material, sj-pdf-1-vdi-10.1177_10406387261460834 for Evaluation of the Sidekick point-of-care progesterone lateral-flow assay for use in equine reproductive management by Sara H. McDowell, Andrew McLain, Luca Moore, Craig J. Ledgerwood, Elizabeth Bailie and Tara Moore in Journal of Veterinary Diagnostic Investigation
