# Supplementary material for: Evaluation of the Sidekick point-of-care progesterone lateral-flow assay for use in equine reproductive management
Source: J Vet Diagn Invest. 2026 Jul 8:10406387261460834. Online ahead of print. doi: 10.1177/10406387261460834 (PMC13346099; doi:10.1177/10406387261460834)
Supplement: sj-pdf-1-vdi-10.1177_10406387261460834 – Supplemental material for Evaluation of the Sidekick point-of-care progesterone lateral-flow assay for use in equine reproductive management [file sj-pdf-1-vdi-10.1177_10406387261460834.pdf]

JVDI: Supplemental material

McDowell SH, et al. Evaluation of the Sidekick point-of-care progesterone lateral-flow assay for use in equine reproductive management

Supplemental Table 1. Progesterone concentration of the 19 equine samples measured by lateral-flow assay (LFA), radioimmunoassay (RIA), and chemiluminescence immunoassay (CLIA).

Subject	Mean LFA concentration, ng/mL	Mean CLIA concentration, ng/mL	RIA concentration, ng/mL
1	4.5	6.3	7.2
2	2.5	3.5	5.2
3	4.0	5.4	7.2
4	4.0	6.1	7.1
5	3.0	4.3	3.4
6	3.5	3.0	3.8
7	6.0	8.0	8.0
8	4.5	4.8	5.7
9	7.5	8.0	8.0
10	7.5	8.0	8.0
11	2.0	2.0	2.0
12	8.0	7.9	8.0
13	6.0	8.0	8.0
14	6.0	7.3	8.0
15	7.0	8.0	8.0
16	7.5	8.0	8.0
17	5.0	7.8	7.8
18	8.0	8.0	8.0
19	8.0	8.0	8.0

Supplemental Table 2. Sensitivity of Deming regression to the assumed measurement-error ratio (λ) for comparison of lateral-flow assay (LFA) with radioimmunoassay (RIA) and chemiluminescence immunoassay (CLIA).

Comparison	Measurement-error ratio, λ	Deming regression slope	Deming regression intercept
LFA vs. RIA	0.5	1.16	−2.42
	1	1.08	−1.87
	2	1.00	−1.33
	4	0.944	−0.928
	6.32	0.918	−0.754
	8	0.908	−0.687
	16	0.889	−0.556
LFA vs. CLIA	0.5	0.630	0.986
	1	0.611	1.12
	2	0.598	1.22
	3.19	0.592	1.26
	4	0.590	1.27
	8	0.585	1.30
	16	0.583	1.32

Supplemental Table 3. Cross-reactivity assessment of altrenogest with lateral-flow assay (LFA).

Altrenogest concentration, μg/mL	LFA reported progesterone concentration, ng/mL		
	Replicate 1	Replicate 2	Replicate 3
1	<2 ng/ml	<2 ng/ml	<2 ng/ml
1	<2 ng/ml	<2 ng/ml	<2 ng/ml
1	<2 ng/ml	<2 ng/ml	<2 ng/ml
